# Fluctuations of Histone Chemical Modifications in Breast, Prostate, and Colorectal Cancer: An Implication of Phytochemicals as Defenders of Chromatin Equilibrium

**DOI:** 10.3390/biom9120829

**Published:** 2019-12-05

**Authors:** Marek Samec, Alena Liskova, Lenka Koklesova, Veronika Mestanova, Maria Franekova, Monika Kassayova, Bianka Bojkova, Sona Uramova, Pavol Zubor, Katarina Janikova, Jan Danko, Samson Mathews Samuel, Dietrich Büsselberg, Peter Kubatka

**Affiliations:** 1 Clinic of Obstetrics and Gynecology, Jessenius Faculty of Medicine, Comenius University in Bratislava, 03601 Martin, Slovakia; marek.samec@gmail.com (M.S.); alenka.liskova@gmail.com (A.L.); koklesova.lenka@gmail.com (L.K.); jan.danko@uniba.sk (J.D.); 2 Department of Histology and Embryology, Jessenius Faculty of Medicine, Comenius University in Bratislava, 03601 Martin, Slovakia; veronika.mestanova@uniba.sk; 3 Department of Medical Biology and Biomedical Center Martin, Jessenius Faculty of Medicine, Comenius University in Bratislava, 03601 Martin, Slovakia; maria.franekova@uniba.sk; 4 Department of Animal Physiology, Institute of Biology and Ecology, Faculty of Science, Pavol Jozef Safarik University, 04001 Kosice, Slovakia; monika.kassayova@upjs.sk (M.K.); bianka.bojkova@upjs.sk (B.B.); 5 Biomedical Center Martin, Jessenius Faculty of Medicine, Comenius University in Bratislava, 03601 Martin, Slovakia; sona.uramova@uniba.sk; 6 OBGY Health & Care, Ltd., 01026 Zilina, Slovakia; prof.pavol.zubor@gmail.com; 7 Department of Pathological Anatomy, Jessenius Faculty of Medicine, Comenius University in Bratislava, 03601 Martin, Slovakia; 8 Department of Physiology and Biophysics, Weill Cornell Medicine in Qatar, Education City, Qatar Foundation, Doha 24144, Qatar; sms2016@qatar-med.cornell.edu

**Keywords:** breast cancer, prostate cancer, colorectal cancer, epigenetics, posttranslational chemical modifications, histone, phytochemicals

## Abstract

Natural substances of plant origin exert health beneficiary efficacy due to the content of various phytochemicals. Significant anticancer abilities of natural compounds are mediated via various processes such as regulation of a cell’s epigenome. The potential antineoplastic activity of plant natural substances mediated by their action on posttranslational histone modifications (PHMs) is currently a highly evaluated area of cancer research. PHMs play an important role in maintaining chromatin structure and regulating gene expression. Aberrations in PHMs are directly linked to the process of carcinogenesis in cancer such as breast (BC), prostate (PC), and colorectal (CRC) cancer, common malignant diseases in terms of incidence and mortality among both men and women. This review summarizes the effects of plant phytochemicals (isolated or mixtures) on cancer-associated PHMs (mainly modulation of acetylation and methylation) resulting in alterations of chromatin structure that are related to the regulation of transcription activity of specific oncogenes, which are crucial in the development of BC, PC, and CRC. Significant effectiveness of natural compounds in the modulation of aberrant PHMs were confirmed by a number of in vitro or in vivo studies in preclinical cancer research. However, evidence concerning PHMs-modulating abilities of plant-based natural substances in clinical trials is insufficient.

## 1. Introduction

Throughout history, natural substances were widely used to treat various diseases [1]. Food of plant origin is associated with health-profitable benefits [2] related to bioactive compounds [3]. Phytochemicals are bioactive non-nutrients found in fruit, vegetable, or other plant sources exerting antioxidant, antiinflammatory, and other beneficial effects [4,5]. Importantly, a combination of phytochemicals in whole foods may be more protective against carcinogenesis due to their additive or synergistic effects [6,7]. Recently, natural substances gained great interest through their anticancer potential mediated via an ability to affect various cancer-associated signaling pathways [8] in different stages of carcinogenesis [1]. Above all, natural compounds exert antiproliferative, proapoptotic, antiangiogenic, antimutagenic, and overall genoprotective abilities [4,9,10,11]. Furthermore, antineoplastic efficacy of plant natural compounds can be mediated via epigenome modulatory mechanisms maintaining gene expression, DNA damage, or repair mechanisms [12]. In addition to the regulation of miRNA [13,14,15,16] expression or DNA methylation [14,16,17,18], plant natural compounds may also regulate posttranslational histone modifications (PHMs) [19].

According to the Global cancer statistics 2018 (GLOBOCAN), breast cancer (BC), prostate cancer (PC), and colorectal cancer (CRC) are included in the five most frequently diagnosed cancer cases as well as the ten most common causes of cancer death representing approximately 5.2 million new cases and 1.8 million deaths in both sexes worldwide. In men, PC and CRC represent the second and third most commonly diagnosed malignant diseases and the fourth and fifth most common causes of cancer-related death worldwide. Additionally, BC followed by CRC represents the most commonly diagnosed cancer type in women with BC and CRC being the first and third main cause of cancer death worldwide [20]. Synthetic therapeutics that target epigenetic modulations of chromatin are already in clinical testing. Several chromatin modulating synthetic drugs, such as entinostat, romidepsin, phenylbutyrate, or their combinations with conventional therapeutics, were found to be potentially effective against BC, PC, and CRC [21,22,23].

An evaluation of alterations of PHMs associated with diverse types of diseases represents the generation of novel clinical approaches that could be used as predictive and prognostic markers for patients [24]. The application of individual patterns of histone modifications, mainly acetylation and methylation of histones H3 and H4 and their amino acid, residuesserve as perspective tools that bridge personalized medicine and epigenetics [25,26]. Advances in preclinical and clinical cancer research constantly indicate important anticancer activities of plant-derived compounds in cancer chemoprevention and treatment [6,9,10,14,27,28,29,30]. Therefore, this review summarizes anticancer effectiveness of dietary phytochemicals, either isolated or mixtures, mediated via their abilities to modulate PHMs associated with PC, BC, and CRC, which are included among the most common cancer types in women and men.

### 1.1. Aim of the Study

The review focuses on the possible role of plant-based naturally occurring compounds (phytochemicals) in chemoprevention and cancer suppression through the modulation of PHMs associated with cancer initiation, progression, invasion, and metastasis. The main aim of the review is to summarize the preclinical and clinical research of BC, PC, and CRC focusing on phytochemicals (isolated or mixtures) and their impact on the modulation of acetylation and methylation resulting in alterations of chromatin structure, and thus, in regulation of transcription activity of the genome.

### 1.2. Source of the Data

Data from the available biomedical English language literature were analyzed and reviewed from the PubMed database. Relevant studies were retrieved by using terms such as “histone” and “posttranslational modifications”, “methylation”, “acetylation”, “phytochemicals”, “natural substances”, or “epigenetic” as either a keyword or medical subject heading (MeSH) term/phrases in searches of the PubMed bibliographic database. We focused on the most recent publications from the year 2015 to 2019.

## 2. Histone Modifications as Molecular Regulators of Chromatin Structure

The nucleus of eukaryotic cells contains DNA packaged into chromatin [31]. The state of chromatin determines an accessibility of DNA to transcriptional machinery and thus, controls gene expression [32,33]. Nucleosome, a basic unit of chromatin, is composed of 147 base pairs of long DNA wrapped around the histone octamer containing two dimers of H2A/H2B and a tetramer of H3 and H4 [34,35]. In addition, histone H1 functions as a linker of the octamer structure [36]. Each core histone contains a globular core and unstructured N-terminal tails [37,38], defined as regions comprised of approximately 25–40 amino acids that are strongly positively charged [39]. Therefore, an affinity between DNA backbone and negatively charged neighboring nucleosomes exists [38]. Despite that the packaging is important in the protection of eukaryotic cells’ genome [38], it also prevents cells’ access to DNA [38]. However, cells apply highly regulated mechanisms to alleviate DNA accessibility in chromatin [40,41]. N-tail domains are in fact associated with various PHMs [39] that influence the structure, folding, and function of chromatin, which consequently affect biological processes, such as expression or repression of target genes, DNA repair, or chromosome condensation [31,42]. Due to the dynamic nature of chromatin, histone tails can also be applied as binding and signaling areas for remodeling and regulatory proteins or as docking sites for various regulators promoting gene transcription [33,34]. PHMs include acetylation and methylation as well as phosphorylation, ubiquitination, sumoylation, adenosine diphosphate (ADP)-ribosylation, etc. [34,37]. In general, PHMs modulate the charge between DNA and histones, thus affecting the structure of the chromatin and transcriptional processes. Moreover, PHMs may also be associated with recognition modules for specific binding proteins [34].

Histone acetylation occurs on highly conserved lysine residues [39]. Importantly, the process of histone acetylation is regulated by the opposite action of two enzyme families, including histone acetyltransferases (HATs) and histone deacetylases (HDACs) [42,43]. Actually, the transfer of acetyl moiety to the ε-amino group of lysine’s side chains is maintained by HATs using acetyl coenzyme A (CoA) as a cofactor. Consequently, lysine´s positive charge is neutralized, leading to the weakening of DNA–histone interactions [43] and enabling transcriptional regulatory proteins to assess chromatin connected to gene activation. On the contrary, HDACs function in the removal of acetyl groups in histones [44], which restores lysine´s positive charge and represses transcription [43]. A balance between HDACs and HATs maintains the chromatin structure and mediates the state of its activity [42]. Above all, histone acetylation is associated with the transcriptional activity of chromatin [33].

Histone methylation is a widely known posttranslational modification involving transfer of a methyl group from a high-energy enzymatic donor to amino groups [33]. Primarily, histone methylation occurs on lysine or arginine residues [32], mainly on H3 or H4 tails [33]. Mono-, di-, or tri-methylation of lysine are located on their ε-amino group and mono-symmetrical dimethylation or asymmetrical dimethylation of arginines occurs on their guanidyl group [32,45]. Depending on the localization, histone methylation can affect transcription positively or negatively [46]. Histone methylation is regulated by methylation modifiers, including histone methyltransferases (HMTs) and histone demethylases (HDMs). Unlike acetylation, histone methylation does not change the charge of histones [45]. Histone methylation induces structural changes influencing the folding of chromatin through an electrostatic mechanism [33] and may also function as a docking site for specific binding proteins, also known as histone readers [45].

Histone phosphorylation is a dynamic posttranslational modification occurring on serine, threonine, and tyrosine residues of core histones, predominantly on N-terminal tails [33]. Histone phosphorylation is maintained by kinases and phosphatases. A transfer of the phosphate group from ATP to the hydroxyl group of a target amino-acid side chain is mediated via kinases. Subsequently, the chromatin structure is influenced by a negative charge added to the histone [43].

ADP-ribosylation is catalyzed by ADP-ribosyltransferases (ARTs) [47], in which single or multiple ADP-ribose units are transferred from nicotinamide adenine dinucleotide to a target protein [48] with simultaneous release of nicotinamide [49]. Histones are considered as one of the most important acceptors of ADP-ribosylation [50]. While mono-ADP-ribosylation occurs mostly outside the nucleus, poly-ADP-ribosylation is found predominantly on nuclear proteins [33].

**Figure 1:**
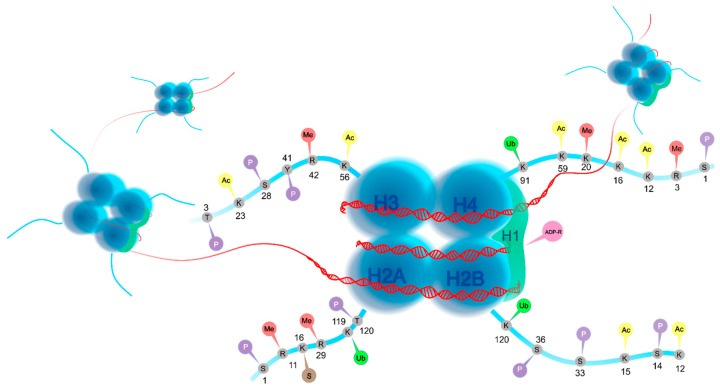
Overview of histone modifications. Modifications of certain amino acids including phosphorylation (P, purple), methylation (Me, red), acetylation (Ac, yellow), ADP-ribosylation (ADP-R, pink), sumoylation (S, brown), and ubiquitination (Ub, green) [33,57,58,59,60,61,62,63,64,65,66,67,68,69,70,71,72,73,74,75].

Furthermore, histones can be modified by conjugation of small proteins, including ubiquitin or a small ubiquitin-like modifier (SUMO) [51]. Histone ubiquitination is a result of an action of three enzyme activities (E1-activating enzyme, E2-conjugating enzyme, and E3-ligase) leading to the formation of an isopeptide bond between carboxyterminal glycine of 76 amino-acids protein ubiquitin, and the ε-amino group of a target lysine residue on the carboxyterminal tail of histone [52,53,54]. Importantly, ubiquitination is related to transcriptional activation or repression depending on the genomic context [54]. Moreover, sumoylation of histones is defined as conjugation of SUMO-1 or 2/3 family to all core histones, histone H1, and histone variants H2A.Z and H2A.X [55]. Similarly, sumoylation may affect gene transcription positively or negatively [56]. All mentioned PHMs are illustrated in Figure 1.

### 2.1. Histone-Modifying Enzymes: Insight into the Regulatory Processes of Acetylation and Methylation

As mentioned above, histone modifications are common mechanisms of PHM proteins representing methylation, acetylation, ubiquitination, ADP-ribosylation, sumoylation, and phosphorylation. These fundamental epigenetic events regulate the expression of genes associated with all aspects of cellular functions [76]. Processes of acetylation and methylation as well as alterations of their enzymatic activity are currently strong prognostic and predictive signatures of cancer progression [77,78,79,80]. Histone acetylation and methylation are two dominant enzymatic interventions of lysine, arginine, or histidine residues of histones such as H3 and H4. These enzymatic modifications are reversible and responsible for the transcriptional repressive or transcriptional active state of chromatin’s structure [81].

#### 2.1.1. Histone Acetyltransferases

HATs and HDACs are two crucial enzyme families directly connected to N-terminal residues of lysine. As described above, HATs are enzymes responsible for the transfer of the acetyl group on lysine residues of histones from acetyl-CoA donors. Depending on localization, HATs are further divided into cytoplasmatic HAT1 and the nuclear fraction of enzymes. According to the mechanism of action and sequence homology, nuclear HATs are subdivided into five classes: GNAT (PCAF, GCN5, ELP3), p300/CBP (CBP, p300), MYST (MOZ, TIP60, HBO1, HMOF, MORF), and fungal Rtt109 family [82]. The enzymatical activity of HATs is regulated via molecular pathways representing events such as participation of the binding partners, autoacetylation of enzymes, and modulation of HAT regulatory domains [83].

#### 2.1.2. Histone Deacetylases

HDACs have a dominant role in the removal of acetyl groups from histone residues. Currently, HDACs have been classified into four subclasses based on the similarity of sequences [84]. According to similarity with the amino-acid sequence of yeast Rpd3 enzymes, class I includes HDAC 1, -2, -3, and -8. Class II is composed of HDAC 4, -5, -6, -7, and -9, that share a sequence similarity with yeast HdaI deacetyltrasferase. Class III represents enzymes such as SIRT 1, -2, -3, -4, -5, -6, and -7, with sequence analogy to yeast Sir2 deacetyl enzymes. HDAC 11 is the only one member of class IV that has high sequence relation to enzymes from class I and class II [84]. HDACs are frequently regulated by several processes affecting enzymes of transcription and posttranscription as well as translation and posttranslation. Molecular pathways associated with modulation of the above-mentioned HDACs represent several ways linked to the addition and/or removal of the phosphoryl group of HDACs of all classes representing the most well-studied area regulating enzymatic functionality [85]. Another manner in which HDACs are regulated by the molecular machinery of cells reflects the interaction of protein–protein, leading to increased enzymatic activities, or this multi-subunit complex can act as a suppressor of HDAC functionality [86,87]. Furthermore, molecular events, including control of gene expression, alteration in splicing of RNA, regulatory impact of miRNA, or availability of cofactors, are directly associated with regulation of HDACs [85,88,89].

#### 2.1.3. Histone Methyltransferases

HMTs represent a number of enzymes with the effector function as transfer catalyzators of methyl groups from methyl donor S-adenosyl methionine (SAM) on arginine and lysine residues in targeting proteins. Nowadays, there is evidence that around 70 enzymes act as catalyzators of methylation of histone amino acid residues [90]. The first HMT, Su(var)3-9 (SUV39H1), was identified in humans as well as in mice. Similarly, SUV38H1 is highly conserved through evolution from yeast to humans [91]. According to the presence of conserved domains, HMTs are divided into three families, including enzymes with the SET domain, enzymes with the Dot1 domain connected to lysine methylation, and the PRMT (protein arginine methyltransferase) class associated with methylation of arginine [32,90]. Molecular mechanisms participating in the modulation of HMTs activity represent events such as posttranscriptional modifications via the ubiquitin-proteasome pathway leading to the degradation of HMT [92]. Another way of HMTs posttranscriptional regulation represents a molecular cascade resulting in phosphorylation of enzymes (e.g., AKT-mediated phosphorylation of EZH2) and thus, in modulation of the catalytic activity of methyltransferases [93]. Importantly, regulatory processes mediated by ncRNA, including miRNA (miR-101) and lncRNA, suggest a linkage between levels of these RNA and the enzymatic activity of HMTs [94,95,96]. Histone methylation is a dominant modification responsible for chromatin remodeling mediated by numerous HMT enzymes. Moreover, the process of demethylation has an equally important role in the regulation of epigenome, and thus, it is directly connected to the modulation of numerous cellular events [97].

#### 2.1.4. Histone Demethylases

Analogically to HMTs, numbers of demethylase enzymes contribute to the removal of the methyl-group from histones. To date, more than 30 HDMs have been identified. The majority of HDMs have JmjC-domain-containing proteins that confer substrate specificity and catalytic functions as demethylase, and only two LSD1 and LSD2 are original demethylases specific for lysine that lack the JmjC-domain [98]. Regulation of demethylases activity is possible via posttranscriptional as well as posttranslational mechanisms. There are several ways in which demethylase activity is modulated. The ubiquitin proteasomal system is one of the major regulation processes responsible for degradation of histone-modifying enzymes. Polyubiquitination and subsequent degradation of enzymes through proteasome is associated with Jumonji domain (JMJD)-2A demethylase [99]. Another regulatory process responsible for the alteration of enzymatic activity of HDMs is phosphorylation mediated by protein kinase A, resulting in the activation of plant homedomain finger protein 2 (PHF2) demethylase [100].

**Figure 2:**
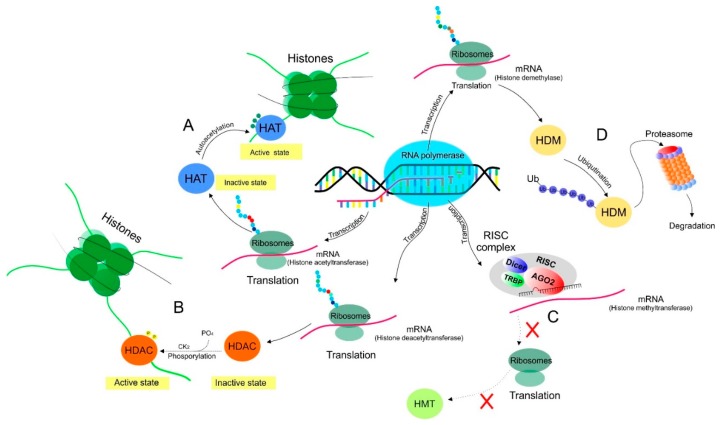
Mechanisms regulating histone-modifying enzymes activity. Specific ways in which the enzymatic activity of histone-modifying enzymes can be regulated are summarized below. (**A**) Schematic figure of autoacetylation when specific amino-acid (K274) in HAT (MYST-family) is unacetylated. In this way, an enzymatic activity of HAT is blocked. The situation when a specific amino acid becomes acetylated changes the conformation and generates a hydrogen bond with other amino acids (S303), resulting in substrate (histone) binding. (**B**) The example of HDAC phosphorylation in which protein kinase CK2 phosphorylates a specific amino acid with a crucial role for enzymatic activity. (**C**) The third scheme represents the regulatory activity of miRNA in a complex RISC (RNA-induced silencing complex) associated with the downregulation of HMT. Recent evidence suggests a linkage between the downregulation of miR-101 and the upregulation of enhancers of zeste homolog 2 (EZH2) methyltransferase in cancer. (**D**) Regulation of HDM activity is also possible via ubiquitination and subsequent degradation in the proteasome. In this manner, the HDM enzyme (JMJD2A) is polyubiquitylated through the activity of complex E3 ligase, resulting in proteasomal degradation.

In summary, all above-mentioned molecular events leading to the modulation of the activity of numerous HDACs, HATs, HMTs, and HDMs associated with chromatin remodeling represent only a few of the multiple ways in which their effector functions can be regulated. Therefore, further investigations in the field of signaling pathways or ncRNA expression as regulatory factors of the catalytic activity of the epigenetic machinery focusing on histones are needed. A detailed overview of selected regulatory pathways controlling the activity of enzymes associated with acetylation and methylation are shown in Figure 2.

### 2.2. Global Patterns of Acetylation and Methylation in Cancer Diseases

**Figure 3:**
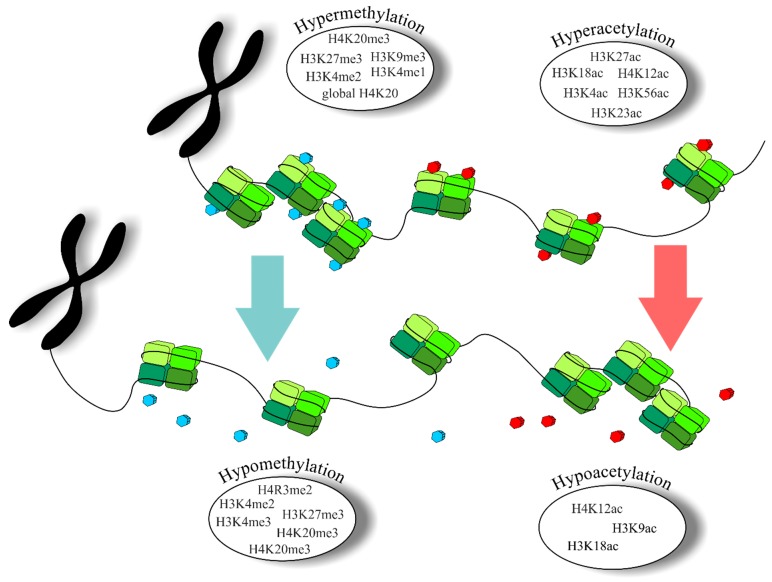
Hypermethylated/hypomethylated and hyperacetylated/hypoacetylated chromatin with specific patterns detected in breast cancer (BC), prostate cancer (PC), and colorectal cancer (CRC). The upper image represents chromatin with increased levels of methylation (compacted chromatin with blue dots) and acetylation (relaxed chromatin with red dots). The lower image illustrates structural events associated with decreasing of methylation and acetylation mediated by HDMs and HDACs. Explanatory notes: Arrows indicate alterations of chromatin structure (green, alteration in methylation pattern; red, alteration in acetylation pattern).

Disequilibrium of catalytic activity of enzymes and thus alterations of PHMs patterns are directly connected to BC, PC, and CRC initiation and promotion [34,101,102,103]. Therefore, individual patterns of histone modifications might be implicated as markers of response to treatment and their specific motifs can correlate with cancer recurrence and overall survival of patients [104,105]. Table 1 summarizes the most frequent variants of histone acetylation and methylation in BC, PC, and CRC, focusing on their specific positions in histones tail and impact on cancer development. Alterations of chromatin’s structure influenced by methylation and/or acetylation associated with BC, PC, and CRC is shown in Figure 3.

## 3. Dietary Phytochemicals Regulating Epigenetic Mechanisms

Phytochemicals are chemical compounds derived from vegetable, fruit, beans, or grains and have many benefits for human health. Moreover, the consumption of plant-derived food may lead to inhibition or elimination of initiation, progression, and development of cancer in in vitro and in vivo models [117] via several mechanisms such as antioxidant, antineoplastic, and antiangiogenic efficacy [13], as well as novel mechanisms based on epigenetic modifications, which play an essential role in the regulation of normal cellular functions [118,119,120]. Epigenetic states of genes have reversible potential and can be changed by intrinsic and extrinsic factors [121]. Several preclinical and clinical studies showed that phytochemicals have an ability to revert abnormal epigenetic modifications, especially PHMs, in different types of cancer such as BC, PC, and CRC.

### 3.1. Impact of Phytochemicals on Histone Chemical Modifications in Clinical and Preclinical Research Focusing on Breast, Prostate, and Colorectal Cancer

#### Breast Cancer

Several papers have described the effects of phytochemicals on PHMs in BC. *Salvia miltiorrhiza*, also known as Danshen, is a traditional Chinese plant characterized by a presence of tanshinone I (T1). T1 represents one of three major diterpene compounds and is the most potent anticancer agent of Danshen. The anticancer potential of T1 evaluated in an in vitro study was associated with the Aurora A gene [122], which is frequently overexpressed in various malignancies including BC [122,123,124]. Generally, Aurora kinases (Aur) are involved in processes of cell division, and AurA plays an important role in chromosomal distribution. Importantly, overexpression of Aurora A in BC is suggested to be related to histone acetylation. Treatment of BC cells with T1 led to the significant decrease in acetylation levels of H3 that was subsequently associated with the downregulation of Aurora A. Therefore, T1 inhibited cancer growth in several BC cell lines (MCF-7, MDA-MB-231, SKBR3, MDA-MB-453) in vitro, at least partially by affecting the function of this gene [122]. Triple-negative breast cancer (TNBC), which represents an aggressive BC subtype with poor prognosis, is highly associated with mutations of tumorsupressor BRCA1 [125,126,127]. However, quercetin and curcumin (CUR) dose-dependently inhibited cell survival and migration of TNBC cell lines in vitro via modulation of BRCA1 expression. The authors of the study concluded that these synergistically acting natural compounds repressed the silencing of BRCA1 via an increase in H3 lysine acetylation of its promoter [127]. Moreover, anticancer efficacy of crystal lapiferin derived from the traditional Algerian plant, *Ferulaves ceritensis*, was evaluated in human BC cells. Consequently, apoptosis-inducible abilities of lapiferin were mediated via several mechanisms, including induction of histone acetylation in MCF-7 cells [128]. Considering that HDAC is overexpressed in various cancer types, extract of *Thymus serpyllum* dose-dependently inhibited HDAC enzyme activities as well as mRNA levels of HDAC1 in MDA-MB-231 cells [129]. Furthermore, our group recently described the chemopreventive abilities of plant natural substances mediated via various mechanisms, including modulation of epigenetic modifications. Clove buds administered in diet significantly increased H4K20me3 and H4K16ac [30] and *Thymus vulgaris* decreased H3K4me3 in a rat model of chemically induced mammary carcinogenesis [14]. Importantly, all these changes represent positive impacts on epigenetic modifications in mammary carcinoma. Moreover, resveratrol (RES) restrained suppressive state of critical tumorsupressors including BRCA1, p53, and p21 in BC cell lines, MCF-7 and MDA-MB-231, which led to inhibition of cancer growth. RES restored the function of the above-mentioned genes via a decrease in repressive methylation marks (H4R3me2s, H3K27me3) and an increase in marks of activating acetylation modifications (H3K9ac, H3K27ac) in histones surrounding promoters of these genes [130]. Similarly, combinatorial proanthocyanidins (GSPs) and RES treatment led to the inhibition of BC cells, which can be affected by various mechanisms including induction of apoptosis or epigenetic intervention, such as a reduction of HDAC activity in MDA-MB-231 and MCF-7 cells [131]. Additionally, a combinatory treatment by sulforaphane (SFN) and Withaferin A (WA), a natural compound from Indian cherry, led to the downregulation of HDAC expression at multiple levels in both MCF-7 and MDA-MB-231 cell lines. The authors concluded that the decreasing trend in HDAC expression is at least partially associated with an ability of SFN and WA to decrease cell viability and induce apoptosis in both cell lines [132]. Similarly, the same combinatorial dietary compounds inhibited cell cycle progression in MCF-7 and MDA-MB-231 cells via downregulation of pRB, CDK4, and Cyclin D1 levels, and an increase in levels of E2F mRNA and p21 independently of p53, while these results occurr simultaneously with an increase in unrestricted histone methylation [133]. Thymoquinone (TQ), a phytochemical found in *Nigella sativa*, also known as black cumin [134], exerted an ability to attenuate the global HDAC activity demonstrated via in silico findings corroborating with in vitro analysis of MCF-7 cells. Moreover, downstream effects of HDAC inhibition by TQ included the induction of proapoptotic gene Bax, a decrease in antiapoptotic Bcl-2, reactivation of HDAC target genes p21 and Maspin, and cell cycle arrest at G2/M phase [135].

Regarding clinical trials, the bioavailability and chemopreventive efficacy of SFN were evaluated in a double-blinded, randomized controlled trial conducted on 54 women scheduled for a breast biopsy. Women were randomized to placebo group and a group administered with Glucoraphanin (GFN), a supplement providing SFN. Importantly, the decrease in peripheral blood mononuclear cell HDAC activity was observed in the supplement group. Moreover, a significant decrease in the level of tissue biomarker HDAC 3 in the supplement group may be associated with reduction of total HDAC activity. However, there was no increase in H3K18ac or H3K9ac in the supplement group. Interestingly, the authors observed a decrease in H3K9ac in ductal carcinoma in situ (DCIS) issue among the placebo group, which could function as a marker of cancer progression. Above all, despite the fact that GFN supplementation for a short period is safe, results of this 2–8 week study are not sufficient to evaluate changes in breast tissue tumor biomarkers [136].

### 3.2. Prostate Cancer

The antitumor effects of naturally occurring compounds of plants in prostate carcinogenesis via regulation of histone modifications are supported by several preclinical studies. SFN is isothiocyanate regulating epigenetic modifications, including histone-tail modifications modulating interactions of DNA-histone. SFN intervention decreased HDAC enzyme activity, whereby H3 acetylation was increased at the promotor region, resulting in higher expression of p21 associated with rapid acetylation of tubulin in PC cells. Moreover, in PrEC (normal) cells, SFN treatment was accompanied only with a short-term decrease of HDAC activity [137]. A study revealed that the impact of SFN on PC cells demonstrated down-regulation of HDAC 6 expression in LNCaP and VCaP PC cells, resulting in destabilization of the androgen receptor that play a crucial role in PC development [138]. Increased telomerase activity was detected as a marker of poor prognosis in numerous neoplastic diseases, including PC. Recently, Abbas et al. evaluated the impact of SFN on the expression of human telomerase reverse transcriptase (hTERT) through the regulation of epigenetic mechanisms in two PC cell lines (LNCaP and DU-145). The results suggested an indirect linkage between the application of SFN and changes in the level of HDAC, resulting in the suppression of hTERT activity in PC cells [139]. Moreover, SFN demonstrated antitumor ability as the regulator of histone modifications associated with the repression of cancer in tramp C1 cells via restoring of Nrf2, the key player in the antioxidant defense. Phytochemical therapy inhibited expression of HDAC 1, -4, -5, and -7, while acetylation of H3 was significantly increased. Results revealed that SFN exerts anticancer potential as an epigenetic regulator in the Nrf2 activating pathway [140]. In addition, Myzak et al. reported that the administration of SFN in PC-3 xenografts in male nude mice resulted in a decrease of HDAC activity in the xenograft, prostate, and mononuclear blood cells [141]. Furthermore, epigallocatechin-3-gallate (EGCG) represents bioactive compound of plants contributing to the regulation of events connected to PHMs in PC. Tissue inhibitor of metalloproteinases-3 (TIMP3) is associated with the acceleration of cancer invasiveness and the development of metastasis. Deb et al. focused on the analysis of modulation of matrix metalloproteinases (MMPs) and reactivation of TIMP3 via epigenetic modifications, such as alterations of histones modifying enzyme activities. PC cell lines DUPRO and LNCaP were treated with green tea polyphenols (GTPs) and EGCG. Phytochemicals intervention in PC cells was accompanied by increased expression of TIMP3, while levels of enhancers of zeste homolog 2 (EZH2) and H3K27me3 marker were significantly reduced. On the other hand, levels of H3K9ac and H3K18ac were higher after treatment with GTPs and EGCG. In the clinical trial, tested patients undergoing proctectomy consumed polyphenon E (1,3g), a GTPs formulation primarily consisting of EGCG, as four capsules per day for up to six weeks, which led to positive epigenetic changes (more about the clinical trial below) [142]. Apigenin (API) is a dietary flavonoid with a plethora of benefits for human health. Treatment of PC cells (PC-3 and 22Rv1) with API led to the reduction of enzymatic activity of HDAC 1 and HDAC 3 on both protein and mRNA levels. Oral administration of API (20 and 50 µg per day/8 weeks) also decreased HDAC 1 and -3 expression and p21/waf1 (associated with regulation of cell cycle arrest) re-expression in the mice PC-3 xenografts model in lower (20 µg) as well as in higher (50 µg) API concentration [143]. Furthermore, API exerted HDACi (inhibitor of HDAC) effect on PC-3 and DU145. The application of API resulted in the reduction of HDAC 1 activity and subsequent acetylation of Ku-70, leading to a dissociation of interaction with Bax followed by induction of apoptosis [144]. Moreover, genistein (GEN) was documented as a phyto-substance affecting epigenetic pathways at the histone level. LNCaP and PCE cells were treated with 5Aza-C and GEN. For both cell lines, the authors revealed changes in levels of acetylation of histones H3 and H4 and increased levels of H3K4me2, H3K4me3, and HAT activity in response to 5Aza-C and GEN. The intervention of phytochemical led to the reactivation of tumor suppressor gene BTG3, which is silenced in many cancer types, including PC. The analogical effects of GEN compared to 5Aza-C (phase II clinical trial) predicted GEN as a potential novel therapeutic drug for patients with PC [145]. Interestingly, CUR was documented as a regulator of many epigenetic pathways [15,146]. Zhao et al. evaluated the impact of CUR on suppression of LNCaP cells via inhibition of the c-Jun-N-terminal kinase (JNK) signaling pathway. Acquired data suggested a decreased level of H3K4me3 in PC cells connected to the reduction of the JNK pathway [147]. Importantly, mixtures of phytochemicals in plants and their synergic effects showed antioxidant and antineoplastic properties in cancer. *Paederia foetida* (PF) is a traditional herb associated with the promotion of male vitality. The authors of the study used PC-3 and DU-145 cells treated with an alcohol extract of PF leaves. An aim of the experiment was an evaluation of the efficiency of plant in epigenetic modifications. Tested PC cells had a lower level of HDAC 1 and HDAC 2 expression after the application of the extract of PF leaves, lupeol and β-sitosterol, resulting in apoptosis, depression of viability, and suppression of cancer cells migration [148].

As discussed above, aberrant modifications of histones are marks of PC development. Numerous studies focused on alterations of the catalytic activity of histone-modifying enzymes induced by phytochemicals in preclinical research and only a few analyzed their impact within a clinical approach. As described above, Deb et al. analyzed the amount of TIMP3 in plasma samples after polyphenon E treatment in patients in the period between tumor biopsy and radical prostatectomy. HDAC 1 activity, EZH2, and trimethylations in H3K27 were reduced in GTPs supplemented prostate tissue [142]. Similarly, SFN demonstrated significant modulation properties via the regulation of PHMs in the clinical study, in which the level of HDAC activity was evaluated in healthy volunteers. In humans, consumption of a single-dose (68 g) of BroccoSprouts with a high level of SFN glucosinate caused repression of HDAC activity in peripheral blood mononuclear cells [141]. Moreover, several studies evaluating an impact of dietary phytochemicals on PC are still in progress or results have not been published yet (NCT02095717; NCT02064673; NCT01265953).

#### Colorectal Cancer

There are several studies that showed alterations of histone acetylation or methylation, which were associated with anticancer activities of phytochemicals. Glycerol trihexanoate, also known as tricaproin (TCN), acquired from chloroform extract of *Simorouba glauca* leaves, demonstrated anticancer activity in the CRC model [149]. TCN induced apoptosis through the reduced oncogenic HDAC 1 activity in HCT-116 and HCT-15 cells. Additionally, in a time- and dose-dependent manner, TCN inhibited the growth of CRC cells but not the growth of normal BEAS-2B cells [150]. Another study indicated the anticancer activity of benzoic acid and its derivatives belonging to the group of phenolic acids commonly found in fruit and vegetable [151]. Dihydroxy benzoic acid (DHBA) decreased HDAC expression, leading to inhibition of cell growth, induction of reactive oxygen species (ROS), and subsequently, to apoptosis in HCT-116 and HCT-15 cells ex vivo and in vitro [152]. An extract of 4β-hydroxywithanolide E (4HWE) from the plant *Physalis peruviana* (Solanaceae) has extensive medicine purposes with potential in oncological research [121]. Treatment by lower concentrations of 4HWE inhibited the growth of HT-29 cells and induced G0/G1 cell cycle arrest. 4HWE at higher concentrations promoted histone chemical modifications and apoptosis. Histone alterations were accompanied with an increased level of SIRT1 in the nucleus, resulting in decreased acetylation in H3K9 and inhibition of c-Jun activity in HT-29 cells [153]. SFN and related isothiocyanates (ITCs) from cruciferous vegetable demonstrated beneficial effects on human health, including cancer disease [154]. ITCs and SFN, in a dose- and time-dependent manner, inhibited HDAC 3 and HDAC 6 activity connected to enhanced acetylation, DNA damage, and degradation of repair proteins, such as CtIP in HCT-116 cells [155]. Moreover, an in vivo study revealed that the administration of SFN in diet increased the acetylation of H3 and H4, inhibited HDAC activity, and suppressed tumorigenesis in mice [156]. A recent study indicated that structural heterocyclic analogs of SFN, i.e., tetrazole side-chain analogs 3D, 8D, and 9D, also affected HAT/HDAC activities, changed histone acetylation status, and reduced HDAC 3 expression, lysine acetyltransferase 2A (KAT2A/GCN5), and P300/CBP-associated factor (PCAF) in HCT-116 cells. Structural heterocyclic analogs of SFN were more effective than SFN in this model. In addition, SFN and its structural analogs (6-SFN and 9-SFN) demonstrated the decreased HDAC 3 expression and increased pH2AX levels as a marker of DNA damage in the model of polyposis in rat colon (Pirc) [157]. Compound K is a metabolite of saponins isolated from ginseng, which downregulated HDAC 1 activity via increased acetylation of histones H3 and H4, leading to cell cycle arrest and induction of apoptosis in human HT-29 cells [158]. Furthermore, a common flavonoid, luteolin (LUT), derived from fruit, vegetable, or herbs, demonstrates anticancer activities connected to the inhibition of cell invasion, transformation, metastasis or angiogenesis, and induction of cell cycle arrest and apoptosis [159]. LUT decreased protein levels of HDACs in HCT-116 cells and suppressed cell proliferation and transformation in HCT-116 and HT-29 cells. Besides that, the decreased methylation of Nrf2 promoter region by LUT induced its downstream antioxidative stress pathway [160]. Moreover, CUR, the main component of *Curcuma longa*, reduced protein expression of HDACs, especially HDAC 4, -5, -6, and -8 in HT-29 cells. Additionally, the oncostatic effect of CUR was linked with other epigenetic modifications—demethylation and upregulation of tumor suppressor gene DLEC1 (lung and esophageal cancer 1) [161]. Furthermore, intraperitoneally administration of extract from an ornamental plant *Alcea rosea* (ARE) reduced the tumor growth in HCT-116 colon cancer cell xenograft due to the loss of EZH2 expression. Also, ARE targeting CSC stemness showed inhibitory effects on the Wnt/β-catenin and Notch signaling pathways. The mechanism of Wnt/β-catenin pathway is supposed to be regulated epigenetically by EZH2 [162]. Another study evaluated TQ with known antioxidant, antiinflammatory, and antineoplastic effects in vitro and in vivo [163,164]. In addition, TQ suppressed HDAC 2 activity and induced histone hyperacetylation in HT-29 cells. Inhibition of tumor growth was related to an increased level of apoptosis in colon cancer xenografts after TQ administration [165].

**Figure 4:**
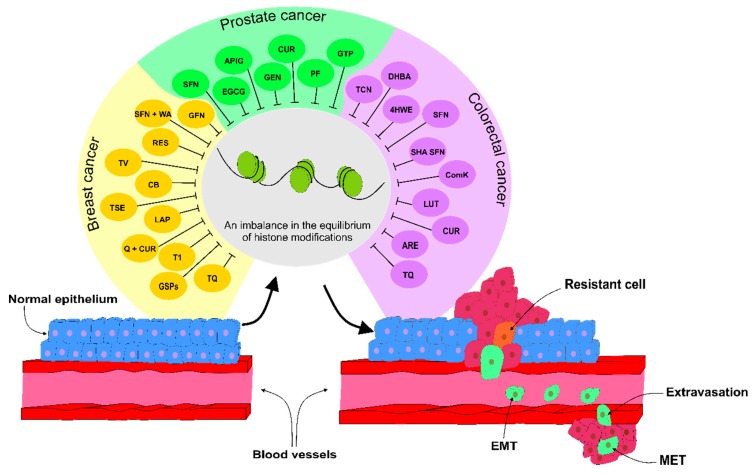
Dietary phytochemicals with an ability to inhibit abnormal modifications of histones leading to carcinogenesis in preclinical and clinical approaches. Throughout cancer development, imbalance in epigenetic modifications, especially PHMs, plays a critical role in the malignant transformation of normal epithelium leading to the cancer progression and metastases. Dietary phytochemicals (mentioned above) positively influenced PHMs reverting abnormal aberrations associated with BC, PC, and CRC. Abbreviations: T1, Tashinone I; Q + CUR, Quercetin and Curcumin; LAP, Lapiferin; TSE, *Thymus serpyllum* extract; CB, Clove buds; TV, *Thymus vulgaris*; RES, Resveratrol; GSPs, proanthocyanidins; SFN + WA, Sulphoraphane and Withaferin A; GFN, Glucoraphanin; SFN, Sulphoraphane; EGCG, Epigallocatechin-3-gallate; API, Apigenin; GEN, Genistein; CUR, Curcumin; PF, *Paederia foetida*; GTPs, Green tea polyphenols; TCN, Tricaproin; DHBA; Dihydroxy benzoic acid; 4HWE, 4β-hydroxywithanolide E; SHA SFN, Structural heterocyclic analogs of sulphoraphane; ComK, Compound K; LUT, Luteolin; ARE, *Alcea rosea* extract; TQ, Thymoquinone.

Table 2 shows an overview of anticancer activities of plant natural compounds in BC, PC, and CRC mediated via modulation of histone modifications. The dietary phytochemicals mentioned above demonstrated anticancer activities via epigenetic alteration, specifically PHMs in different types of cancer including BC, PC, and CRC (Figure 4). Despite many preclinical studies that were positively related to reversion of abnormal histone modification via phytochemicals in cancer processes, there were only few clinical trials demonstrating only limited data and conclusions for clinical oncologists. 

## 4. Conclusions and Future Directions

Carcinogenesis and metastatic cancer comprise both genetic and epigenetic elements. Global modifications in epigenetic characteristics in the cell chromatin are unambiguously recognized as a hallmark of cancer. Based on comprehensive research, epigenetic mechanisms such as DNA methylation, non-coding RNAs, nucleosome positioning, or histone chemical modifications demonstrate categorical linkage with the carcinogenesis. Importantly, the covalent posttranslational chemical modification of histone proteins is proven as a mechanism that plays an important role in the chromatin remodeling, and consequently, in the regulation of numerous genes’ expression that may be strongly associated with different aspects of carcinogenesis [166,167]. From the clinical point of view, PHMs induced by targeted therapy may represent an effective tool for better management of cancer disease.

Extensive cancer research in the last decade demonstrates that beneficial epigenetic changes can be induced therapeutically or via changes in dietary habits [168]. Plant-derived bioactive molecules (phytochemicals) are of particular interest within oncological research. Numerous phytochemicals or natural mixtures of plant compounds present in whole foods show significant antitumor properties via multiple cell signaling pathways and mechanisms, and thus, represent perspective and potentially effective tools for chemoprevention and targeted therapy of cancer disease [14,30,169,170,171,172]. Moreover, an administration of dietary phytochemicals is the way of a cost-effective and readily applicable clinical approach in the management of cancer, including the most commonly occurring BC, PC, and CRC. On the contrary to the genetic (inborn, non-modifiable) components of carcinogenesis, epigenetic changes that are particularly important for the development of sporadic cases of cancer (BC, PC, and CRC comprise 75–90% of all cases) are strongly associated with environmental and lifestyle risk factors, including eating habits [173,174,175]. This emerging knowledge leads to considerable interest in nutri-epigenetics or nutri-epigenomics, which focuses on the influence of dietary compounds on epigenetic mechanisms [176]. This approach has gained considerable attention because epigenetic changes are reversible and/or modifiable. Extensive oncological research demonstrated that plant natural compounds may be effective in targeting epigenetic alterations associated with the cancer promotion and progression as well as the primary chemoprevention by affecting the carcinogenesis in early stages during initiation [167]. A great amount of phytochemicals exerted anticancer activities via modulation of PHMs, as was demonstrated mainly in in vitro as well as in vivo preclinical studies focusing on BC, PC, and CRC [14,118,133,141,142,146,157,160,172]. However, the usefulness of phytochemicals within clinical approaches is not sufficiently investigated in this topic.

Targeting of histone-modifying enzymes by phytochemicals or whole plant substances (foods) that will be able to restore the expression of specific genes to normal levels, and thus induce apoptosis or decrease proliferation, metastatic spreading, and oxidative stress in transformed cells, represents a challenge for preclinical and clinical oncologists. Despite the fact that the exact nature of mechanisms by which phytochemicals act is not fully understood, the application of plant natural substances represent a perspective clinical approach, e.g., in increasing the sensitivity for standard anticancer therapy or application in the cancer chemoprevention setting [177]. Moreover, the assessing of plant-derived modifiers of histones’ chemical changes regarding the cancer stem cells survival that is associated with the relapse and multidrug resistance may provide useful data for clinical oncologists. In addition, coming studies could be directed toward the improved bioavailability of plant-derived chromatin modulators by utilizing, for example, nanoparticles carriers. Importantly, future studies need to be targeted more towards a better understanding of mechanisms that affect histone-modifying enzymes and increasing the potency of these plant bioactive molecules against cancer. For the discovery and development of new effective phytochemicals or their mixtures, novel molecular targets need to be investigated to achieve a detailed understanding of the specific chromatin atlas in numerous cancer cell lines and tissues with different genotypes and phenotypes. In this regard, the full mechanistic understanding of the complexity of the epigenetic network is a crucial challenge for investigators that can open new and fundamental progress in this area [166]. In addition, a deeper understanding of the global patterns of PHMs and their consequences may reveal important molecular targets for dietary phytochemicals that can be clinically applied as modern weapons against cancer. Unlike DNA methylation status and RNA interference analyses that are realized through standard methodologic techniques, certain questions remain about the histone code that needs a major breakthrough in advances of peptides/proteins separation methods [168]. However, impressive achievements in biomedicine methodology in the last years make for an optimistic scenario in this regard.

Novel research data demonstrate the variability of histone chemical modifications in individual cells within primary cancer mass, and thus uncover a new dimension of tumor heterogeneity. Differences among cancer cells within tumor tissue is observed in the level of acetylation and methylation of specific histone residues. As mentioned, epigenetic heterogeneity in cells is significantly related to the clinical outcome of cancer patients and cancer risk individuals [178]. Multiomic cancer diagnostics, including analyses of epigenetic fluctuations, progressive screening programs, and individualized patient profiling and stratification, are demands important for clinical practice that allow and facilitate personalized predictive and preventive clinical advancements in individuals [179,180,181]. The appropriateness of specific phytochemicals/whole plant substances as an “epi-drugs“ against cancer is already experimentally well-established and has considerable potential to commence a new area of individualized approaches in the medical practice management and oncological research [182]. Synthetic therapeutics that target epigenetic modulations of chromatin have shown ambiguous results within clinical testing so far. However, their combination with plant-derived chromatin modulators may potentially improve the positive effects of histone chemical changes and thus, enhance the general efficacy of conventional therapeutics in cancer disease. In this regard, the complex measures of epigenetic biomarkers have a great potential to improve the overall cancer management (including BC, PC, and CRC) in favor of predictive, preventive, and personalized medical healthcare and can be assumed as the “proof-of principle” model for their potential use in other multi-factorial diseases and genetic predispositions.

## Figures and Tables

**Table 1:** Unique chromatin signatures and their impact on cancer.

Type of Cancer	Type of Study	Histone Modification	Effect	References
**Acetylation**				
BC	clinical trial (n = 880)	*↓ H3K9ac; *↓ H3K18ac; *↓ H4K12ac	poor prognostic BC subtypes (basal carcinoma, HER-2+)	[105]
	clinical trial (n = 121)	↑ H3K23ac	shorter overall survival	[106]
	in vitro (MCF10A, MCF7, MDA-MB-231)	↑ H3K4ac	progression from initial transformation to aggressive metastatic phenotypes	[107]
PC	LNCaP/C4-2 cells	↑ H3K18ac	progression from hormone-sensitive to castrate resistant PC	[77]
	clinical trial (n = 71)	↑ H3K18ac	↑ risk of metastasis and PCs recurrence	[78]
	clinical trial (n = 279)	↑ H3K18ac	↑ 1.71-fold increased risk of PCs recurrence	[108]
CRC	clinical trial (n = 80)	global acetylation of H3	poor overall survival	[109]
	clinical trial (n = 12)	↑ H3K27ac	regulation of genes with changeable expression	[110]
	retrospective study (n = 250)	↑ H4K12ac; ↑ H3K18ac	↑ HDAC2; ↑ progression from adenoma to adenocarcinoma	[111]
	retrospective study (n = 304)	↑ H3K56ac; ↑ H4K16ac	↓ tumor regression; ↑ survival	[112]
**Methylation**				
BC	clinical trial (n = 880)	*↓ H4R3me2; *↓ H3K4me2; H4K20me3; *↓ H4R3me2	poor prognostic BC subtypes (basal carcinoma, HER-2+)	[105]
	in vitro (MDA-MB-231)	↓ H3K4me2; ↓ H3K27me3	↑ invasive and tumorigenic capacity of CSCs	[79]
	clinical trial (n = 112) in vitro (HBL-100, MDA-MB-231, BT-474, MCF-7, MCF10A)	↓ H4K20me3	poor prognosis ↑ invasiveness	[113]
	clinical trial (n = 142)	↓ H3K27me3	↓ overall survival time	[114]
PC	clinical trial (n = 34)	↑ H3K27me3	poor prognosis	[101]
	clinical trial (n = 113)	↑ H3K4me1	↑ risk of recurrence	[115]
	clinical trial (n = 279)	↑ H3K4me2	↑ 1.8-fold increased risk of relapse	[108]
	clinical trial (n = 204)	global methylation H4K20	marker of lymph node metastasis/correlation with Gleason score	[80]
CRC	clinical trial (n = 254)	↑ H4K20me3; ↑ H3K9me3; ↓ H3K4me3	↓ tumor regression; ↑ survival; good prognosis	[116]
	in vitro (DLD-1 cell line); in vivo (BALB/c nude mice)	↑ H3K9me3	↑ cell motility; tumor formation and metastasis	[113]

Explanatory notes: ↑ increase; ↓ decrease; *↓ moderate to low levels; ac, acetylation; me, methylation. Abbreviations: BC, breast cancer; CRC, colorectal cancer; CSCs, cancer stem cells; HDAC, histone deacetylase; HER-2+, human epidermal growth factor receptor 2 positive; PC, prostate cancer.

**Table 2:** Anticancer activities of plant natural compounds mediated via modulation of histone modifications.

Natural Compound	Cancer Type	Study Design	Effects on PHMs	Effects on Cancer Cells	Ref
T1	BC	MCF-7, MDA-MB-231, SKBR3, MDA-MB-453 cells	↓ H3 acetylation	↓ cancer growth	[122]
Q + CUR		MDA-MB-231, MDA-MB-468 cells	↑ BRCA1 histone H3K9 acetylation	↓ survival and migration	[127]
LAP		MCF-7 cells	↑ histone acetylation	↑ apoptosis	[128]
TSE		MDA-MB-231 cells	↓ HDAC	↓ proliferation ↑ apoptosis	[129]
CB		Sprague-Dawley rats	↑ H4K20me3, ↑ H4K16ac	↑ anticancer effects	[30]
TV			↓ H3K4me3	↑ anticancer effects	[14]
RES		MCF-7, MDA-MB-231 cells	↓ H4R3me2s, ↓ H3K27me3, ↑ H3K9ac, ↑ H3K27ac	↓ cancer growth	[130]
GSPs + RES		MDA-MB-231, MCF-7 cells	↓ HDAC activity	↑ apoptosis ↑ anticancer effects	[131]
SFN + WA		MCF-7, MDA-MB-231 cells	↓ HDAC	↓ cell viability ↑ apoptosis	[132]
			↑ unrestricted histone methylation	↓ cancer growth	[133]
TQ		MCF-7 cells	↓ global HDAC activity	↑ apoptosis Reactivation of HDAC target genes (p21, Maspin)	[135]
GFN	Women scheduled for breast biopsy	Double-blind, randomized, placebo-controlled clinical trial; supplement group (n = 27)—~250 mg of a broccoli seed extract. Placebo group (n = 27)—microcrystalline cellulose	↓ HDAC, ↓ HDAC 3	Not sufficient results	[136]
SFN	PC	PC3 cells	↓ HDAC enzyme activity, ↑ H3 acetylation at promotor region for P21	↑ apoptosis ↑ cell cycle arrest	[137]
		LNCaP, VCaP cells	↓ HDAC 6	↓ cancer growth	[138]
		LNCaP, DU-145 cells	↓ HDAC, ↑ H3K18ac	↓ immortality	[139]
		Tramp C1 cells	↓ HDAC 1, -4, -5, and -7, ↑ H3 acetylation	↑ anticancer effects	[140]
		PC-3 xenografts in male nude mice; clinical study (n = 3)	↓ HDAC activity in xenografts and in PBMC of healthy volunteers	↓ cancer growth	[141]
EGCG		DUPRO, LNCaP cells	↓ EZH2, ↓ H3K27me3 ↑ H3K9ac, ↑ H3K18ac	↓ invasion and migration	[142]
API		PC-3/22Rv1 cells; mice PC-3 xenografts	↓ HDAC 1, -3	↑ apoptosis ↑ cell cycle arrest	[143]
		PC-3/DU145 cells	↓ HDAC 1	↓ cell viability ↑ apoptosis	[144]
GEN		LNCaP, PC-3 cells	↑ acetylation of H3, H4, H3K4me2 and H3K4me3, ↑ HAT activity	↑ anticancer effects	[145]
CUR		LNCaP cells	↓ H3K4me3	↓ cancer growth ↑ apoptosis	[147]
PF		PC-3, DU-145 cells	↓ HDAC 1, -2	↑ apoptosis ↓ cell viability ↓ migration	[148]
GTPs		clinical study (n = 5), patients treated with GTPs in the period between tumor biopsy and radical prostatectomy	↓ HDAC 1, EZH2, and H3K27me3 in GTPs supplemented prostate tissue of patients compared with no treatment group	↓ not sufficient results	[142]
TCN	CRC	HCT-116, HCT-15 cells	↓ HDAC 1	↓ cancer growth	[150]
DHBA		HCT-116, HCT-15 cells	↓ HDAC	↓ cancer growth ↑ apoptosis	[152]
4HWE		HT-29 cells	↑ SIRT1, ↓ H3K9ac	↑ apoptosis	[153]
SFN		HCT-116 cells	↓ HDAC 3, -6	↑ DNA damage	[155]
		APCmin mice	↑ acetylation of H3 and H4, ↓ HDAC	↑ apoptosis ↑ cell cycle arrest	[156]
SHA SFN		HCT-116 cells; model of polyposis in rat colon (Pirc)	↓ HDAC, ↓ KAT2A/GCN5, ↓ PCAF	↑ anticancer effects	[157]
ComK		HT-29 cells	↓ HDAC 1, ↑ acetylation of H3 and H4	↑ apoptosis ↑ cell cycle arrest	[158]
LUT		HCT-116 cells	↓ HDAC	↓ proliferation ↓ transformation	[160]
CUR		HT-29 cells	↓ HDAC 4, -5, -6, -8	↓ cancer growth	[161]
ARE		HCT-116 colon cancer cell xenografts	↓ EZH2	↓ cancer growth	[162]
TQ		HT-29 cells; HT-29 xenografts	↓ HDAC 2, ↑ histone hyperacetylation	↓ cancer growth ↑ apoptosis	[165]

Explanatory notes: ↑ increase; ↓ decrease. Abbreviations: T1, Tashinone I; Q + CUR, Quercetin and Curcumin; LAP, Lapiferin; TSE, *Thymus serpyllum* extract; CB, Clove buds; TV, *Thymus vulgaris*; RES, Resveratrol; GSPs, Proanthocyanidins; SFN + WA, Sulphoraphane and Withaferin A; GFN, Glucoraphanin; SFN, Sulphoraphane; EGCG, Epigallocatechin-3-gallate; API, Apigenin; GEN, Genistein; CUR, Curcumin; PF, *Paederia foetida*; GTPs, Green tea polyphenols; TCN, Tricaproin; DHBA; Dihydroxy benzoic acid; 4HWE, 4β-hydroxywithanolide E; SHA SFN, Structural heterocyclic analogs of sulphoraphane; ComK, Compound K; LUT, Luteolin; ARE, *Alcea rosea* extract; TQ, Thymoquinone; PHMs, posttranslational histone modifications; EZH2,enhancers of zeste homolog 2; HDAC, histone deacetylase; PBMC, peripheral blood mononuclear cell; HAT, histone acetyltransferase; SIRT1, sirtuin1; KAT2A/GCN5, lysine acetyltransferase 2A; PCAF, P300/CBP-associated factor.
